# Neutralizing Antibodies after Infection with Dengue 1 Virus

**DOI:** 10.3201/eid1302.060539

**Published:** 2007-02

**Authors:** Maria G. Guzman, Mayling Alvarez, Rosmari Rodriguez-Roche, Lídice Bernardo, Tibaire Montes, Susana Vazquez, Luis Morier, Angel Alvarez, Ernest A Gould, Gustavo Kourí, Scott B Halstead

**Affiliations:** * “Pedro Kourí” Tropical Medicine Institute, Havana, Cuba; † Instituto Medicina Tropical, Caracas, Venezuela; ‡ Centre for Ecology and Hydrology, Oxford, United Kingdom; § Pediatric Dengue Vaccine Initiative, Bethesda, Maryland, USA

**Keywords:** Dengue, Cuba, dengue 2, neutralizing antibody, American dengue 2 genotype, dengue hemorrhagic fever, research

## Abstract

Severity of disease is markedly increased when infection with dengue virus type 2 (DENV-2) follows infection with DENV-1. Studies have shown that heterologous neutralizing antibody titers are inversely correlated with severity of a second infection. If this mechanism controlled disease severity in Cuba, heterotypic antibody titers should have declined over time. To determine whether phenotypic changes in dengue antibodies occur over time, we analyzed serum samples collected 4–8 and 20–22 years after DENV-1 infection. We found a significant increase in mean titer of homologous DENV-1 neutralizing antibodies and a significant decrease in heterologous antibodies to 1 of 2 genotypes of DENV-2 virus (the American genotype). Asian DENV-2 viruses were not neutralized during either interval; however, the American genotype underwent phenotypic changes in heterotypic viral neutralizing antibodies in the predicted direction. This finding may be related to the time-dependent changes in severity of disease found with secondary dengue infection.

## Abstract 2

Severity of disease is markedly increased when infection with dengue virus type 2 follows infection with dengue virus type 2 by an interval of 20 years.

During 1977, Cuba experienced a nationwide outbreak of dengue fever (DF). More than 500,000 cases caused by a dengue 1 virus (DENV-1) of Southeast Asian origin were reported (*1*,*2*). Seroepidemiologic studies during 1978–1979 demonstrated hemagglutination-inhibition antibodies against dengue virus in 44.46% of the population (*1*). In 1981, an Asian genotype dengue 2 (DENV-2) produced a major islandwide epidemic of DF and dengue hemorrhagic fever (DHF); >400,000 cases were reported, 10,000 of which resulted in DHF and 158 deaths (101 children) from DHF (*3*–*8*). During 1982–1996, strong vector-control programs stopped transmission of dengue viruses.

In 1997, an Asian genotype DENV-2, closely related to the 1981 strain, entered Cuba and circulated in the island’s second largest city, Santiago de Cuba, producing a severe outbreak of DF and DHF (*9*,*10*). At the time of the outbreak, 25%–35% of the population 18–54 years of age were monotypically immune to DENV-1 because of infections during 1977–1979 (*1*,*2*,*11*). During the 1997 epidemic, an estimated 4,810 adults experienced a second dengue infection with DENV-2, 18–20 years after infection with DENV-1 (*10*). Of this group, 205 patients were hospitalized with DHF, 12 of whom died. No cases of DHF or dengue shock syndrome were observed in children (*12*). When standardized for age, case-fatality rates for persons who had been infected with DENV-1 during 1977–1979 and secondarily infected with DENV-2 in 1997 were 3–4× higher than for persons who had secondary DENV-2 infections in 1981 (*13*). In addition, virtually all (≈100%) secondary DENV-2 infections in the 1997 Santiago de Cuba outbreak were clinically overt in marked contrast to primary DENV-2 infections, of which only 3.0% produced overt disease (*10*).

Both DENV-2 viruses, from 1981 and 1997, belong to the same genotype. Although amino acid differences in structural and nonstructural regions of the 2 genomes might contribute to the observed differences in disease severity, the low intrinsic virulence of the 1997 DENV-2 virus was remarkable. In this study, we focused on the possibility that presence or absence of heterotypic neutralizing antibodies might contribute to severity of secondary DENV-2 infections.

Several observations suggest this to be a mechanism for controlling dengue disease severity during heterotypic infections. The first such observations were made by Sabin, who observed a 3-month period of cross-protection to DENV-2 illnesses after DENV-1 infections in human volunteers (*14*). When DENV-1–immune volunteers were challenged with DENV-2 at intervals >3 months, classical DF occurred. A comparable observation was made in a school-based study in Thailand (*15*), in which 40 children experienced predominantly secondary DENV-2 infections; of these, 33 were fully protected from disease accompanying DENV-2 infections and only 7 were hospitalized. Of the former, human monocyte assay of undiluted serum showed that 31 had DENV-2 neutralizing antibodies from a prior heterotypic dengue infection. By contrast, serum from 6 children who had severe disease did not have neutralizing antibodies, but it enhanced DENV-2 infections (*15*). A similar observation was made in Iquitos, Peru, where DENV-1 had been endemic since 1990. In 1995, an American genotype DENV-2 was introduced into this population that was already highly immune to DENV-1 (*16*). Despite large numbers of persons who were infected initially with DENV-1 and subsequently with DENV-2, no DHF cases were observed. Plaque-reduction neutralization test (PRNT) of DENV-1–immune human serum samples obtained in 1994 in Iquitos showed that nearly all contained high levels of neutralizing antibodies to American genotype but not Asian genotype DENV-2 viruses. The latter viruses have circulated for a long time in populations who are immune to multiple dengue viruses and who could plausibly have lost DENV-1–like epitopes by preferential selection of antibody escape mutants (*17*).

Thus, from these 2 studies we deduced that cross-reactive, dengue-neutralizing antibodies may down-regulate secondary dengue infections and prevent enhanced infections while mediating disease in persons with a different immune status. These observations are supported by recent studies in which DENV-1–immune monkeys were challenged with either American or Asian DENV-2 (*18*). On the basis of these observations, we examined whether dengue antibodies undergo phenotypic changes after many years; such changes would help explain the observed increase in disease severity accompanying secondary DENV-2 infections.

## Materials and Methods

### Serum Samples

Serum samples were submitted to our laboratory from a nationwide dengue surveillance program implemented during 1981–1985 (103 samples) and 1999 (2,000 samples). ELISA results showed no evidence of acute dengue infection. To avoid analyzing serum from persons infected by any other DENV, we excluded samples from Santiago de Cuba province because of the DENV-2 epidemic that occurred in 1997 (*19*,*20*).

Serum samples were first tested for dengue immunoglobulin G (IgG) by an ELISA inhibition method that used a DENV-1 antigen shown to provide the same or better sensitivity and specificity as tests that use all 4 dengue viruses. Samples with dengue IgG were retested by PRNT, which used strains of the 4 dengue serotypes, including 2 DENV-2 strains classified as either Asian (*3*,*4*,*8*) or American genotype (*21*) (Table 1). Testing for all dengue viruses by PRNT was conducted on BHK-21, clone 15 cells (*22*,*23*). Serum was diluted to 1:10, and then serial 10-fold dilutions were made in Earle’s minimal essential medium (MEM). To obtain 15–20 plaques in a 24-well tissue culture plate, we mixed 100 µL of each serum dilution with 100 µL of media containing 80 PFUs of the assayed viruses and incubated this mixture at 37°C for 1 h. Then 50 µL of virus-serum mixture was added in triplicate onto 0.5 mL media containing 2.5×10^5^ cells. After incubating this mixture for 4 h at 37°C in an atmosphere of 4.5% CO_2_, we added 0.5 mL of overlay medium that contained 3% medium viscosity carboxymethylcellulose prepared in MEM without phenol red with 10% heat-inactivated fetal bovine serum, 1% glutamine (2 mmol/L), 100 U penicillin, and 100 µg/mL streptomycin. Infected cells were incubated for 5–9 d, depending on the virus serotype (7–9 d for DENV-1 and DENV-3, 5 d for DENV-2, and 6 d for DENV-4), under the same conditions. After incubation, plates were stained with a solution of naphthol blue-black dye and acetic acid, and the plaques were counted. Serum samples were tested simultaneously against each DENV strain; each serum dilution was tested in triplicate.

**Table 1:** Dengue virus strains used in this study

Serotype	Strain	Passage no.*	Place and year of isolation
DENV-1†	Angola	4PC6/36 1PVero 1PC6/36	Angola, 1988
DENV-2‡	A15/81	4PMB 4PC6/36	Cuba, 1981
DENV-2†‡	I348600	4P C6/36	Colombia, 1986
DENV-3	116/00	3P C6/36	Cuba, 2000
DENV-4†	Dominica	7P C6/36	Dominica, 1981

^*^*P, passage; C6/36, *Aedes albopictus* cell line; Vero, green monkey kidney cell line; MB, mouse brain.

†DENV, dengue virus; received from Robert Shope, University of Texas Medical Branch, Galveston, Texas, USA.

‡DENV-2, A15/81 strain (Asian genotype), DENV 2, I348600 (American genotype).

Antibody titers were expressed as the reciprocal of the endpoint dilution. For statistical purposes, samples with a titer <10 were assigned a titer of 5. Calculations of 50% endpoint plaque-reduction neutralization titers (PRNT_50_) were made by using log probit paper and the method of Russell et al. (*24*). According to criteria previously established (*25*), samples with neutralizing antibody titers ≥30 to only 1 dengue virus were considered evidence of primary dengue infection. Considering the epidemiology of dengue in Cuba and using the DENV-2 strain that circulated during the 1981 epidemic, we classified samples that had dengue neutralizing antibodies ≥30 to DENV-1 but <5 for DENV-2 (A15/81 strain), DENV-3, and DENV-4 as a past primary DENV-1 infection during the 1977–1979 epidemic.

### Statistical Analysis

For data analysis, we used GraphPad Prim 2.0 (SPSS Inc., Chicago, IL, USA). Neutralizing antibody titers were expressed as mean titers. Mean titers were compared to detect significant differences between antibody titers to viruses in each studied group and in both groups of samples by using 1-way analysis of variance followed by the Bonferroni multiple comparison test. Statistical significance was defined as p<0.05. The Fisher exact test was used to compare the positive percentages of neutralizing antibody to each virus in each group of samples.

## Results

Of the 103 serum samples collected during 1981–1985 and the 2,000 collected in 1999, dengue IgG antibodies were detected by screening ELISA inhibition method in 50 (48.5%) and 826 (41.3%), respectively. From these, the 50 samples in the first group (group 1) and 89 representative samples from the second (group 2) were classified as monotypic DENV-1–immune serum on the basis of PRNT_50_ results with the 4 dengue serotypes.

Table 2 shows that the geometric mean titer of homologous neutralizing antibodies increased significantly in samples collected after 22 years compared with those collected 4–8 years after the DENV-1 epidemic of 1977. In contrast, over this same period, heterotypic antibodies directed against the American genotype of DENV-2 declined significantly in the number of samples that had heterotypic neutralizing antibodies to this genotype and in geometric mean titer. DENV-1–immune serum obtained years after inapparent infection showed little heterotypic neutralization of Asian DENV 2 (12%), DENV-3 (8%), or DENV-4 (2%) viruses.

**Table 2:** Neutralization of dengue viruses by dengue virus 1–immune serum collected 4–8 years (group 1) and 20–22 years (group 2) after primary infection*

Serotype (strain)	Group 1		Group 2	
	Positivity (%)	GMT	Positivity (%)	GMT
DENV-1 (Jamaica/77)	50 (100)	93	89 (100)	140.6
DENV-2 (A15/81)	6 (12)	5.5	19 (21)	6.5
DENV-2 (I348600)	36 (72)†	30	40 (45)‡	10.2
DENV-3 (116/00)	4 (8)	5.6	9 (10)	5.9
DENV-4 (Dominica)	1 (2)	5.1	13 (15)	6.2

*GMT, geometric mean titer; DENV, dengue virus.

†p<0.00001 compared with DENV-2 (A15/81), DENV-3, and DENV-4 viruses in group 1.

‡p<0.01–0.0001 compared with DENV-2 (A15/81), DENV-3, and DENV-4 viruses in group 2.

The Figure shows each data point, together with mean log_10_ neutralizing antibody titers to the viruses tested in the studied groups. Means of DENV-1 antibodies differed significantly between groups 1 and 2. Means of antibody titers were significantly different (p<0.001) when DENV-2 (I/348600) was compared with DENV-3 and DENV-4 viruses. Significant differences (p<0.001) were also noted in heterotypic neutralization of DENV-2 (I/348600) in samples from groups 1 and 2. Means of DENV-2 (A 15/81), DENV-3, and DENV-4 did not differ between groups 1 and 2.

**Figure:**
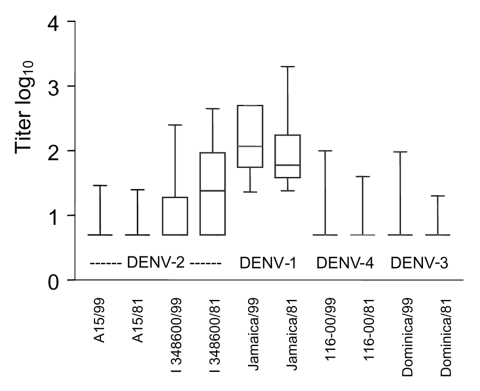
Log_10_ antibody (Ab) titers for human dengue virus type 1–immune serum samples collected in 1999 (89 samples) and 1981–1985 (50 samples, mean).

## Discussion

We present 2 new findings. After DENV-1 infection, homotypic neutralizing antibody titers increase, and heterotypic antibody titers to 1 of 2 genotypes of DENV-2 virus (the American genotype) decrease.

However, our study had several limitations. One problem was that limited serum quantities precluded our ability to test for neutralization and enhancement in primary cultures of human monocytes. Another problem was that the effect of heterotypic neutralizing antibodies on the severity of DENV-2 infections during the 1981 epidemic should have been studied in a representative selection of samples collected before the 1981 outbreak from persons infected by DENV-1 during 1977–1979. Long-term kinetics of neutralizing antibodies requires that samples be collected at intervals from the same persons. To compensate for our inability to conduct longitudinal studies with the same persons, we studied relatively large numbers of samples from randomly selected persons who were immune to DENV-1. Serum available for study was sent to our diagnostic laboratory over a period of many years and stored at –20°C. Samples were sent from representative age and ethnic groups from all geographic areas of Cuba, excluding Santiago de Cuba province. We provide independent confirmation of the cross-neutralization of American genotype DENV-2 by antibodies raised to DENV-1 infections in Cuba.

Consistent with our research hypothesis, we observed a decrease in heterotypic DENV-2 neutralizing antibodies over time. Like Kochel et al. (*17*), we were unable to detect significant heterotypic neutralization to the Asian genotype DENV-2 viruses. We did not observe increases in heterotypic DENV-3 or DENV-4 antibody titers. Unexpectedly, we did observe an increase in titer of homologous DENV-1 antibodies at 4–8 and 20–22 years after infection.

Although we did not detect significant heterotypic neutralization of the A15/81 Asian DENV-2 strain at a 1:10 dilution, these results do not rule out the possibility that neutralization might have been detected at lower dilutions. Limited serum volumes prevented us from testing the panel of DENV-1–immune serum collected during 1981–1985 for heterotypic neutralizing antibodies to the 1997 DENV-2 strain (58/97) isolated during the 1997 Santiago de Cuba epidemic and classified as Asian genotype (*9*). However, we did test group 2 serum (collected in 1999). No differences were observed in neutralization of the A15/81 or 58/97 DENV-2 strains by DENV-1–immune serum at a dilution of 1:10. Only 18% of group 2 samples showed neutralizing activity to the 58/97 DENV-2 strain with a geometric mean titer of 7.1. We believe the antibodies measured in the 2 groups were derived from infections that occurred in Cuba in 1977. Among persons who contributed to each group of serum samples, none had been vaccinated against yellow fever and few had traveled outside Cuba.

Our results demonstrate long-term changes in heterotypic dengue neutralizing antibodies. Although we did not detect neutralization of Asian DENV-2 in vitro, we question whether some degree of neutralization might have occurred in vivo, which might have affected disease severity. For example, the neutralization test is not particularly good at predicting protective immunity. Recently, Endy et al. (*26*) reported that levels of preinfection neutralizing antibodies against DENV-2 (standard strain and virus isolated during illness) were not associated with severity of secondary DENV-2 infection. However, in the same study, higher levels of preexisting neutralizing antibodies against DENV-3 were associated with lower viremia levels and milder disease. Many possible reasons exist for these complexities, including the artificiality of existing dengue viral neutralization tests or differences in ability of antibodies to neutralize different dengue strains of the same genotype.

The first report that heterotypic neutralizing antibodies might be an important mechanism of down-regulating the severity of dengue infection was deduced from the prospective study of school children in Bangkok, Thailand. Children who had heterotypic DENV-2 neutralizing antibodies before they became infected with DENV-2 (their antibodies were predominantly the result of prior DENV-1 infections) experienced only inapparent secondary DENV-2 infections. In contrast, DHF/dengue shock syndrome developed in children whose serum lacked detectable heterotypic neutralizing antibodies (but contained dengue-enhancing antibodies) (*15*). These studies tested undiluted serum, before illness, in elutriated monocytes from donors with no immunity to flaviviruses.

The contemporary explanation of long-term persistence of antibodies after viral infection is based on evidence of the presence of long-lived B memory and plasma cells. The improvement in homotypic neutralizing antibody titer and decrease in heterotypic neutralizing antibody titer described here is reminiscent of affinity maturation. However, long-lived plasma cells would not be expected to participate in the selection process required for affinity maturation. In an earlier study of serum samples from US military personnel with inapparent Japanese encephalitis virus infection, over the 1–5 years after infection, the log neutralization index increased from a mean of 1.7 to 3.5 (*27*). This earlier study and our present study are unique in that they measured qualitative attributes of human antibodies for long intervals after infection with wild-type flavivirus. Our preliminary data suggest a continuous process of selection of populations of dengue virus antibodies with increasing homologous reactivity and a concurrent decrease in heterotypic cross-reactions.

Our results require confirmation and further study. To study antibody titers in the same persons, we will attempt to locate the persons from whom samples were collected during 1981–1985 and collect serum in volumes that may permit tests for dengue-enhancing antibodies and neutralizing antibodies to several dengue strains. Our present results could simply reflect increases and decreases in avidity of antibodies with the passage of time. We plan to investigate this possibility by using the same strains as well as a nondengue flavivirus antigen.

## References

[1] Cantelar de Francisco N, Fernandez A, Albert Molina L, Perez Balbis E. [Survey of dengue in Cuba. 1978–1979]. Rev Cubana Med Trop. 1981;33:72–8. 7034065

[2] Mas P. Dengue fever in Cuba in 1977: some laboratory aspects. In: Proceedings of Dengue in the Caribbean, 1977; 1979 May 8–11; Montego Bay, Jamaica. Washington: Pan American Health Organization; 1979. p. 40–2.

[3] Sariol CA, Pelegrino JL, Martinez A, Arteaga E, Kouri G, Guzman MG. Detection and genetic relationship of dengue virus sequences in seventeen-year-old paraffin-embedded samples from Cuba. Am J Trop Med Hyg. 1999;61:994–1000. 10674684 10.4269/ajtmh.1999.61.994

[4] Alvarez M, Guzman MG, Rosario D, Vazquez S, Pelegrino JL, Sariol CA, [Direct sequencing of an amplified product from a serum sample]. Rev Cubana Med Trop. 1996;48:53–5. 9768271

[5] Guzman MG, Kouri GP, Bravo J, Soler M, Vazquez S, Santos M, Dengue haemorrhagic fever in Cuba. II. Clinical investigations. Trans R Soc Trop Med Hyg. 1984;78:239–41. 10.1016/0035-9203(84)90286-4 6464114

[6] Guzman MG, Kouri GP, Bravo J, Calunga M, Soler M, Vazquez S, Dengue haemorrhagic fever in Cuba. I. Serological confirmation of clinical diagnosis. Trans R Soc Trop Med Hyg. 1984;78:235–8. 10.1016/0035-9203(84)90285-2 6464113

[7] Kouri G, Guzman MG, Bravo J. Hemorrhagic dengue in Cuba: history of an epidemic. Bull Pan Am Health Organ. 1986;20:24–30. 3768589

[8] Guzman MG, Deubel V, Pelegrino JL, Rosario D, Marrero M, Sariol C, Partial nucleotide and amino acid sequences of the envelope and the envelope/nonstructural protein-1 gene junction of four dengue-2 virus strains isolated during the 1981 Cuban epidemic. Am J Trop Med Hyg. 1995;52:241–6. 7694966 10.4269/ajtmh.1995.52.241

[9] Rodriguez-Roche R, Alvarez M, Gritsun T, Rosario D, Halstead S, Kouri G, Dengue virus type 2 in Cuba, 1997: conservation of E gene sequence in isolates obtained at different times during the epidemic. Arch Virol. 2005;150:415–25. 10.1007/s00705-004-0445-1 15578237

[10] Guzman MG, Kouri G, Valdes L, Bravo J, Alvarez M, Vazques S, Epidemiologic studies on dengue in Santiago de Cuba, 1997. Am J Epidemiol. 2000;152:793–9. 10.1093/aje/152.9.793 11085389

[11] Guzman MG, Kouri GP, Bravo J, Soler M, Vazquez S, Morier L. Dengue hemorrhagic fever in Cuba, 1981: a retrospective seroepidemiologic study. Am J Trop Med Hyg. 1990;42:179–84. 2316788 10.4269/ajtmh.1990.42.179

[12] Guzman MG, Alvarez M, Rodriguez R, Rosario D, Vazquez S, Valdes L, Fatal dengue hemorrhagic fever in Cuba, 1997. Int J Infect Dis. 1999;3:130–5. 10.1016/S1201-9712(99)90033-4 10460923

[13] Guzman MG, Kouri G, Valdes L, Bravo J, Vazquez S, Halstead SB. Enhanced severity of secondary dengue-2 infections: death rates in 1981 and 1997 Cuban outbreaks. Rev Panam Salud Publica. 2002;11:223–7. 10.1590/S1020-49892002000400003 12049030

[14] Sabin AB. Research on dengue during World War II. Am J Trop Med Hyg. 1952;1:30–50. 14903434 10.4269/ajtmh.1952.1.30

[15] Kliks SC, Nisalak A, Brandt WE, Wahl L, Burke DS. Antibody-dependent enhancement of dengue virus growth in human monocytes as a risk factor for dengue hemorrhagic fever. Am J Trop Med Hyg. 1989;40:444–51. 2712199 10.4269/ajtmh.1989.40.444

[16] Watts DM, Porter KR, Putvatana P, Vasquez B, Calampa C, Hayes CG, Failure of secondary infection with American genotype dengue 2 to cause dengue haemorrhagic fever. Lancet. 1999;354:1431–4. 10.1016/S0140-6736(99)04015-5 10543670

[17] Kochel TJ, Watts DM, Halstead SB, Hayes CG, Espinoza A, Felices V, Effect of dengue-1 antibodies on American dengue-2 viral infection and dengue haemorrhagic fever. Lancet. 2002;360:310–2. 10.1016/S0140-6736(02)09522-3 12147378

[18] Kochel TJ, Watts DM, Gozalo AS, Ewing DF, Porter KR, Russell KL. Cross-serotype neutralization of dengue virus in *Aotus nancymae* monkeys. J Infect Dis. 2005;191:1000–4. 10.1086/427511 15717278

[19] Kouri G, Guzman MG, Valdes L, Carbonel I, del Rosario D, Vazquez S, Reemergence of dengue in Cuba: a 1997 epidemic in Santiago de Cuba. Emerg Infect Dis. 1998;4:89–92. 9454563 10.3201/eid0401.980111 PMC2627664

[20] Valdes L, Guzman MG, Kouri G, Delgado J, Carbonell I, Cabrera MV, [Epidemiology of dengue and hemorrhagic dengue in Santiago, Cuba 1997]. Rev Panam Salud Publica. 1999;6:16–25. 10.1590/S1020-49891999000600003 10446511

[21] Leitmeyer KC, Vaughn DW, Watts DM, Salas R, Villalobos de Chacon I, Ramos C, et al. Dengue virus structural differences that correlate with pathogenesis. J Virol. 1999;73:4738–47. 10233934 10.1128/jvi.73.6.4738-4747.1999 PMC112516

[22] Fernandez RJ, Vazquez S. Serological diagnosis of dengue by an ELISA inhibition method (EIM). Mem Inst Oswaldo Cruz. 1990;85:347–51. 2134709 10.1590/s0074-02761990000300012

[23] Morens DM, Halstead SB, Repik PM, Putvatana R, Raybourne N. Simplified plaque reduction neutralization assay for dengue viruses by semimicro methods in BHK-21 cells: comparison of the BHK suspension test with standard plaque reduction neutralization. J Clin Microbiol. 1985;22:250–4. 4031038 10.1128/jcm.22.2.250-254.1985 PMC268369

[24] Russell PK, Nisalak A, Sukhavachana P, Vivona S. A plaque reduction test for dengue virus neutralizing antibodies. J Immunol. 1967;99:285–90. 6031202

[25] Guzman MG, Kouri G, Bravo J, Soler M, Martinez E. Sequential infection as risk factor for dengue hemorrhagic fever/dengue shock syndrome (DHF/DSS) during the 1981 dengue hemorrhagic Cuban epidemic. Mem Inst Oswaldo Cruz. 1991;86:367. 1842425 10.1590/s0074-02761991000300011

[26] Endy TP, Nisalak A, Chunsuttitwat S, Vaughn DW, Green S, Ennis FA, Relationship of preexisting dengue virus (DV) neutralizing antibody levels to viremia and severity of disease in a prospective cohort study of DV infection in Thailand. J Infect Dis. 2004;189:990–1000. 10.1086/382280 14999601

[27] Halstead S, Russ SB. Subclinical Japanese encephalitis. II. Antibody responses of Americans to single exposure to JE virus. Am J Hyg. 1962;75:202–11. 13904014

